# Outcomes after total first metatarsophalangeal joint arthroplasty for hallux rigidus: a systematic review and meta-analysis

**DOI:** 10.1186/s42836-026-00433-3

**Published:** 2026-10-02

**Authors:** Karthik Chinnaswamy, Archisha Marya, Anand Pillai

**Affiliations:** 1 Faculty of Biology, Medicine and Health, The University of Manchester, Manchester, M13 9PL UK; 2 Department of Trauma and Orthopaedics, Wythenshawe Hospital, Manchester, M23 9LT UK

**Keywords:** Hallux rigidus, First metatarsophalangeal joint arthroplasty, Range of motion, Patient-reported outcomes, Revision, Meta-analysis, Survivorship

## Abstract

**Background:**

The role of first metatarsophalangeal (MTP) joint arthroplasty in hallux rigidus remains controversial. Although used as a motion-preserving alternative to arthrodesis, outcomes vary between studies and implant designs. This systematic review and meta-analysis evaluated clinical outcomes, range of motion (ROM), and implant survivorship following total first MTP arthroplasty.

**Methods:**

A systematic review identified studies reporting pre- and postoperative outcomes following total first MTP arthroplasty for hallux rigidus. Pooled outcomes were the American Orthopaedic Foot and Ankle Society Hallux Metatarsophalangeal-Interphalangeal (AOFAS-HMI) score, visual analogue scale (VAS) pain score, and ROM. One postoperative timepoint per study was selected using a prespecified rule, prioritising the timepoint closest to 24 months, or follow-up between 12 and 36 months where discrete timepoints were not reported. Random-effects pre-post meta-analysis used an assumed pre-post correlation of 0.5. Heterogeneity, prediction intervals, leave-one-out analysis, and sensitivity analyses were performed.

**Results:**

Twelve studies were included in the AOFAS-HMI meta-analysis, seven in the VAS meta-analysis, and thirteen in the ROM meta-analysis. Arthroplasty was associated with a pooled improvement in AOFAS-HMI of 42.76 points (95% CI 38.44–47.09; *p* < 0.001), a pooled reduction in VAS pain of 5.24 points (95% CI 3.82–6.67; *p* < 0.0001), and a pooled improvement in ROM of 27.02° (95% CI 17.19–36.86; *p* < 0.001). Heterogeneity was high. Prediction intervals remained wholly positive for AOFAS-HMI and VAS, but crossed zero for ROM, indicating less consistent motion gain. Secondary complication outcomes were reported as crude observational frequencies across studies with variable follow-up durations. Radiographic failure occurred in 14.5% of reported feet and revision in 10.0%; these figures are not annualised incidence rates or formal meta-analytic survivorship estimates. Longer follow-up cohorts suggested reduced implant durability over time.

**Conclusions:**

Total first MTP arthroplasty is associated with improvement in pain and function, but these benefits should be interpreted alongside appreciable revision and radiographic failure rates. ROM improves on average, although restoration of motion remains inconsistent and non-physiological. Current evidence therefore supports total arthroplasty as an option in select patients rather than a uniformly reliable alternative to arthrodesis and highlights the need for further implant development and longer-term evaluation of newer designs.

**Supplementary Information:**

The online version contains supplementary material available at https://doi.org/10.1186/s42836-026-00433-3.

## Introduction

Hallux rigidus is the most common degenerative disorder of the first metatarsophalangeal (MTP) joint and a frequent cause of forefoot pain, gait alteration, and footwear limitation in otherwise active adults [1]. Initial treatment is usually non-operative and may include analgesia, footwear modification, orthoses, and injections. For a substantial proportion of patients, however, these measures provide only temporary relief and operative management is ultimately considered [2].

The aetiology of hallux rigidus is multifactorial, and in many patients no single causative factor is identified. Proposed contributors include previous trauma, familial predisposition, anatomical predisposition, anatomical variation of the first MTP joint, altered foot biomechanics, and first-ray instability. Family history and female gender have also been associated with an increased risk of bilateral cases [2].

Surgical options for advanced hallux rigidus include cheilectomy, periarticular osteotomy, arthrodesis, and joint replacement [3]. Arthrodesis remains the benchmark procedure for advanced disease because it provides reliable pain relief and durable functional outcomes. However, fusion abolishes motion at the first MTP joint and may alter gait mechanics, plantar-flexion requirements, and footwear choices, which can be relevant to active patients and those who prioritise preservation of joint motion [4].

Total first MTP arthroplasty has been developed as a motion-preserving alternative intended to relieve pain while maintaining joint mobility and toe length. Earlier implant generations, particularly silicone designs, were associated with concerns regarding osteolysis, loosening, and failure [5]. More recent metallic and ceramic implants have shown encouraging short-term improvements in pain and function, but concerns remain regarding radiolucency, subsidence, progressive loosening, and revision with longer follow-up [6].

Prior reviews and meta-analyses have tended to focus on the choice between arthrodesis and arthroplasty, often pooling heterogeneous implants and fair-to-poor quality studies, with limited quantitative synthesis of patient-reported outcomes and survivorship for total arthroplasty [5, 7, 8].

There remains uncertainty regarding what patients can realistically expect from contemporary total first MTP arthroplasty in terms of pain relief, functional improvement, motion gain, and implant durability. The aim of the present systematic review and meta-analysis was therefore to synthesise postoperative pain, function, and range of motion (ROM) outcomes after total first MTP arthroplasty for hallux rigidus, and to characterise revision, failure, and complication patterns across implant generations.

## Methods

This systematic review was completed in accordance with the Preferred Reporting Items for Systematic Reviews and Meta-Analyses (PRISMA) guidelines.

### Search strategy

A systematic literature search was performed in MEDLINE, Embase (via Ovid), and the Cochrane Library. The search was carried out on 4 May 2025 and covered studies published between January 1990 and May 2025. Search terms included combinations of terms relating to the first metatarsophalangeal joint, hallux rigidus, osteoarthritis, and arthroplasty, including the terms first metatarsophalangeal joint, first MTPJ, big toe, first toe, hallux rigidus, osteoarthritis, and arthroplasty.

Study selection was undertaken in two stages by two independent reviewers. Titles and abstracts were screened first, followed by full-text review of potentially eligible studies. Disagreements were resolved through discussion, with adjudication by a third senior reviewer where required.

### Eligibility criteria

Studies were eligible for inclusion if they:

1. included adults aged 18 years or older with hallux rigidus or degenerative change of the first metatarsophalangeal joint.
2. evaluated total first metatarsophalangeal joint arthroplasty.
3. included a study population of at least 10 feet.
4. used a randomised controlled, cohort, or case series design; and
5. reported a mean follow-up of at least 12 months and at least one prespecified clinical, functional, survivorship, or complication outcome.

Studies were excluded if they:

1. were published in a language other than English.
2. were cadaveric studies.
3. were case reports, reviews, meta-analyses, conference abstracts, or poster abstracts.
4. evaluated hemiarthroplasty, interpositional arthroplasty, or silicone/silastic implants; or
5. reported duplicate or overlapping patient populations, in which case the study with the longest follow-up was retained.

Where comparative studies included both eligible and non-eligible treatment arms, only the data from the eligible total arthroplasty cohort were extracted for qualitative and quantitative synthesis.

### Primary outcomes

The primary quantitative outcomes were postoperative change in function, pain, and range of motion. Function was assessed using the American Orthopaedic Foot and Ankle Society Hallux Metatarsophalangeal-Interphalangeal (AOFAS-HMI) score, pain using the visual analogue scale (VAS), and motion using range of motion (ROM) in degrees. These outcomes were selected because they reflect the principal intended benefits of total first MTP arthroplasty: pain reduction, functional improvement, and preservation of motion.

### Secondary outcomes

Secondary descriptive outcomes included revision, implant survivorship, prosthetic loosening, infection, implant failure, persistent pain, non-revision reoperation, and radiographic failure. Implant survivorship was assessed descriptively using revision as a surrogate marker of implant failure. These outcomes were selected to provide a broader assessment of the safety and durability of first metatarsophalangeal joint arthroplasty and to identify factors that may influence longer-term clinical outcome and implant survivorship.

### Data extraction and management

Data extracted included study characteristics, including author, year of publication, and country of origin; cohort characteristics, including sample size, patient demographics, and duration of follow-up; and all prespecified primary and secondary outcomes. Data extraction was performed independently by two reviewers, with any discrepancies resolved through discussion and, where necessary, by a third senior reviewer.

### Data synthesis

Continuous outcomes were synthesised separately by outcome domain. The prespecified primary outcome families for quantitative synthesis were function, pain, and range of motion, represented by AOFAS-HMI, VAS, and ROM in degrees, respectively. Outcomes were analysed in separate meta-analyses to avoid pooling non-equivalent constructs or scales and to allow comparison of the magnitude and consistency of postoperative improvement across domains.

For each study, one postoperative timepoint per outcome was selected for the primary analysis. Where multiple discrete postoperative timepoints were reported, the timepoint closest to 24 months was selected. Where outcomes were reported only at mean follow-up rather than at a discrete postoperative interval, studies were eligible for inclusion in the primary synthesis if mean follow-up was between 12 and 36 months. Studies reporting follow-up outside this range were not included in the primary pooled analyses but were retained for prespecified sensitivity analyses.

All quantitative syntheses were conducted as within-group preoperative to postoperative meta-analyses of continuous outcomes. For functional score and ROM analyses, improvement was defined as postoperative minus preoperative mean score. For pain analyses, improvement was defined as preoperative minus postoperative mean score such that positive values consistently represented clinical improvement across all pooled analyses.

Because the included studies were predominantly single-arm pre-post designs, preoperative and postoperative values were treated as paired observations. Study-level effect estimates were calculated as raw mean change scores. For all study outcomes, standard deviations of change were derived from the reported preoperative and postoperative standard deviations using the standard formula for paired data.

$$SDchange= \sqrt{{SDpre}^{2}+{SDpost}^{2}-2r(SDpre)(SDpost)}$$

where *r* represents the within-person pre-post correlation coefficient. A value of *r* = 0.5 was applied uniformly across all primary analyses. Sensitivity analyses were prespecified using alternative correlation coefficients of *r* = 0.3 and *r* = 0.7 to assess the robustness of the pooled estimates to this assumption.

Study-specific sampling variances were calculated from the derived change-score standard deviations and sample sizes. Pooled estimates were synthesised using inverse-variance weighted random-effects meta-analysis. Random-effects models were fitted using restricted maximum likelihood estimation. Hartung-Knapp adjustment was applied to provide more robust confidence intervals given the modest number of studies available within each outcome-specific synthesis.

Between-study heterogeneity was quantified using Cochran’s Q statistic, τ^2^, and I^2^. In addition, 95% prediction intervals were calculated for each pooled analysis to estimate the likely dispersion of true effects in future comparable clinical settings.

Prespecified sensitivity analyses were undertaken to assess the robustness of the primary findings. These included re-estimation of pooled effects using alternative assumed pre-post correlation coefficients, inclusion of studies outside the primary follow-up window, and inclusion of studies with methodological features likely to reduce comparability in the primary analysis, such as broader outcome definitions. Sensitivity analyses were interpreted in relation to changes in pooled effect size, confidence intervals, heterogeneity, and prediction intervals.

Exploratory subgroup analyses and meta-regression according to implant material, prosthesis design, and follow-up duration were considered. However, ceramic implants were represented in only 2 of 12 studies in the AOFAS-HMI analysis, 2 of 7 studies in the VAS analysis, and 3 of 13 studies in the ROM analysis. Given the sparse and imbalanced subgroup sizes, formal subgroup analyses and meta-regression were considered underpowered and potentially misleading and were therefore not undertaken.

Influence and robustness were further examined using leave-one-out analyses, Baujat plots, and influence diagnostics, including externally standardised residuals, DFFITS, Cook’s distance, leverage, and deletion diagnostics. Forest plots were generated for each primary meta-analysis to display study-level and pooled effects.

All statistical analyses were performed in R using the metafor package.

### Quality assessment

Risk of bias was assessed using the Risk of Bias in Non-Randomised Studies of Interventions tool (ROBINS-I) for non-randomised studies and the Cochrane Risk of Bias 2 tool (RoB 2) for any randomised controlled trials. Two reviewers independently evaluated risk of bias, with disagreements resolved through discussion or consultation with a third reviewer.

## Results

### Study selection

The literature search identified 1,543 records, of which 512 duplicates were removed, leaving 1,031 records for title and abstract screening. Following screening, 990 records were excluded, and 41 full-text articles were assessed for eligibility.

A total of 32 studies were included in the qualitative synthesis. Of these, 15 studies provided sufficient data for inclusion in at least one quantitative meta-analysis. The number of studies contributing to each outcome-specific meta-analysis varied according to outcome reporting and data availability, with 12 studies contributing to AOFAS-HMI, 7 to VAS, and 13 to ROM analyses.

**Fig. 1:**
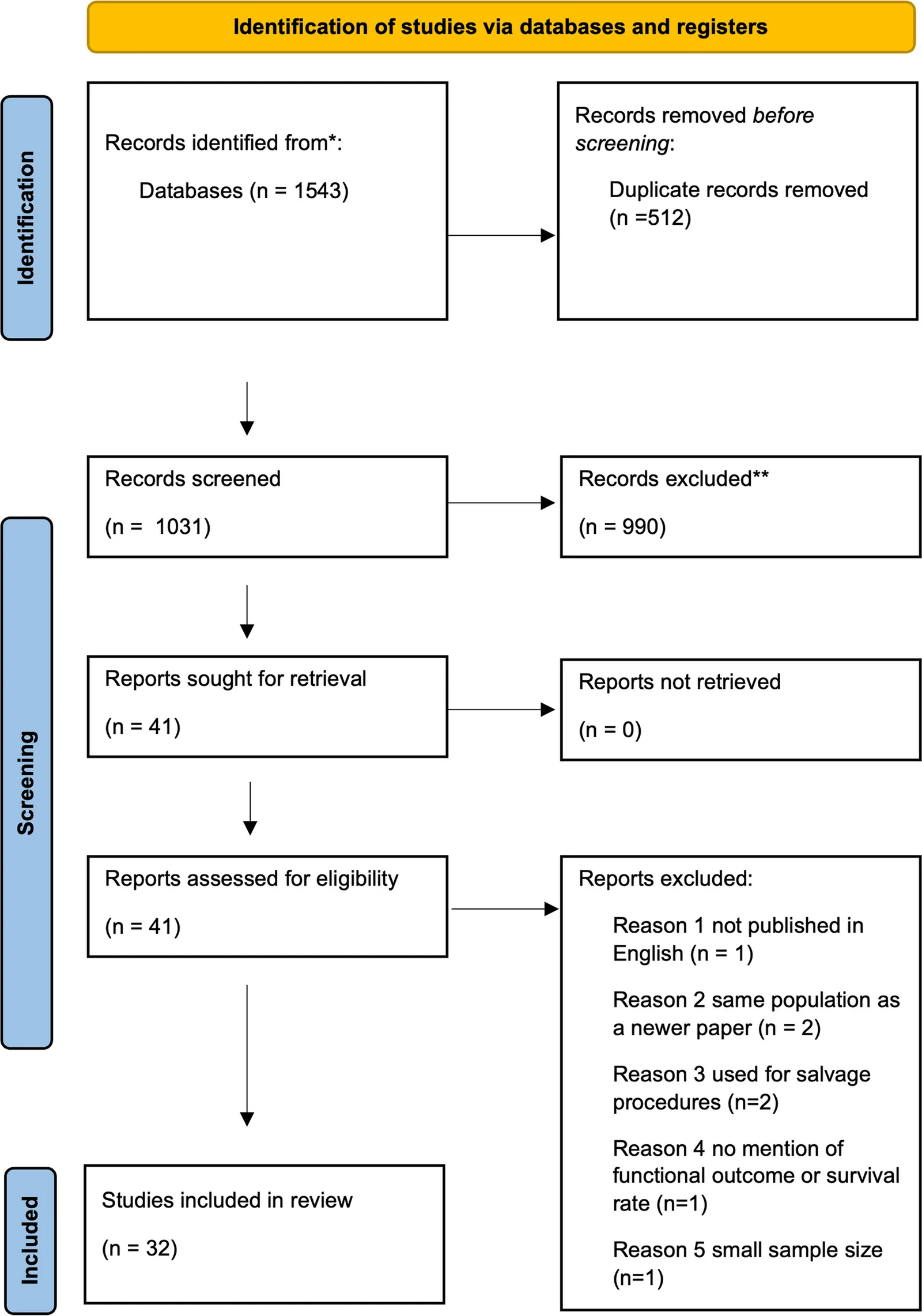
PRISMA diagram

Studies were excluded from quantitative synthesis where variance data were not reported or could not be derived, where outcomes were reported only postoperatively without preoperative values, or where outcome definitions or follow-up reporting were not compatible with the prespecified analytic framework. The study selection process is summarised in the PRISMA flow diagram (Fig. 1).

### Study characteristics

A total of 32 studies were included, encompassing 960 patients (1,074 feet). Study characteristics are summarised in Table 1.

**Table 1:** Study and baseline characteristics of included studies

Author, Year, Country	Study design	Patients (feet)	Mean patient age (years)	Gender (M/F)	Mean follow-up (months)	Implant name	Material
Akkurt et al., 2016, Turkey [9]	Retrospective	30(30)	66.2 ± 5.3	6/24	27.5 (range 17–35)	ToeFit-Plus	Metallic
Arbuthnot et al., 2008, United Kingdom [10]	Prospective	42(40)	NR	NR	21 (range 3–36)	MOJE ceramic	Ceramic
Bartak et al., 2022, Czech Republic [11]	Retrospective	18(19)	56.8 ± 7.9	NR	146.4 ± 13	ToeFit-Plus	Metallic
Barwick et al., 2008, United Kingdom [12]	Retrospective	22(24)	54.5 ± 6.3	5/17	26 (range 12–47)	MOJE ceramic	Ceramic
Brewster et al., 2010, United Kingdom [13]	Retrospective	29(32)	56 (range 38–79)	9/20	34 (range 6–74)	MOJE ceramic	Ceramic
Chee et al., 2011, United Kingdom [14]	Retrospective	37(41)	62 (range 50–77)	6/31	33 (range 12–60)	MOJE ceramic	Ceramic
Daniilidis et al., 2010, Germany/Italy/Austria [15]	Prospective	23(23)	57 ± 3.7	7/16	18	ToeFit-Plus	Metallic
Dawson-Bowling et al., 2012, United Kingdom [16]	Retrospective	29(31)	61.9 (range 37–76)	9/21	58 (range 28–97)	MOJE ceramic	Ceramic
Dulgeroglu et al., 2017, Turkey [17]	Retrospective	15(15)	61.6 ± 9.07	3/12	21.7 ± 2.3	Integra Movement Great Toe System	Metallic
Duncan et al., 2014, United Kingdom [18]	Retrospective	57(69)	61 (range 45–83)	12/57	22 (range 6–48)	ToeFit-Plus	Metallic
Erdil et al., 2013, Turkey [19]	Retrospective	12(12)	61.42 ± 7.45	4/8	27.91 (range 24–41)	ToeFit-Plus	Metallic
Erkocak et al., 2013, Turkey [6]	Retrospective	24(26)	55 (range 38–78)	8/16	29.9 (range 25–62)	ToeFit-Plus	Metallic
Fadel et al., 2005, United Kingdom [20]	Retrospective	13(14)	51.3 (range 28–61)	1/12	25.9 (range 20–40)	MOJE ceramic	Ceramic
Gupta et al., 2008, United Kingdom [21]	Prospective	20(20)	57	7/13	12.2 ± 5.4	ToeFit-Plus	Metallic
Gupta et al., 2017, United Kingdom [22]	Retrospective	47(49)	57	19/36	134 (range 84–144)	ToeFit-Plus	Metallic
Hassan et al., 2006, United Kingdom [23]	Retrospective	17(18)	56 (range 36–72)	2/15	18 (range 11–31)	MOJE ceramic	Ceramic
Horisberger et al., 2016, Switzerland [24]	Prospective	25(29)	63.1 ± 10.2	10/15	49.5 ± 7.7	Metis (Integra)	Metallic
Ibrahim et al., 2004, United Kingdom [25]	Retrospective	8(11)	58 (range 51–80.5)	1/7	17 (range 10–22)	MOJE ceramic	Ceramic
Karpe et al., 2018, United Kingdom [26]	Prospective	28(34)	60.5 ± 8.0	14/15	27.7 ± 9.25	Roto-Glide	Metallic
Kofoed et al., 2017, Denmark [27]	Prospective	80(90)	58 ± 5.8	27/53	138 ± 2	Roto-Glide	Metallic
Kundert et al., 2005, Switzerland [28]	Retrospective	14(14)	NR	NR	48 (range 36–63)	ToeFit-Plus	Metallic
McGraw et al., 2010, United Kingdom [29]	Prospective	48(63)	56 ± 10.75	20/43	44 ± 14.75	MOJE ceramic	Ceramic
Mermerkaya et al., 2016, Turkey [30]	Retrospective	19(19)	57.1 ± 5.8	8/11	27.1 ± 7.5	ToeFit-Plus	Metallic
Nagy et al., 2014, United Kingdom [31]	Retrospective	24(31)	55 ± 6	0/24	81 ± 27	MOJE ceramic	Ceramic
Omonbude et al., 2004, United Kingdom [32]	Retrospective	13(14)	49.8 ± 9.0	5/8	12.0 ± 1.0	MOJE ceramic	All-ceramic
Pulavarti et al., 2005, United Kingdom [33]	Prospective	32(36)	57 ± 8.5	8/24	47 ± 8.25	Bio-Action	Metallic
Sinha et al., 2010, United Kingdom [34]	Retrospective	14(15)	59	4/10	61 ± 4.25	Bio-Action	Metallic
Stone et al., 2017, United Kingdom [35]	Randomised controlled trial	52(66)	NR	NR	Median 182.4	Biomet/Zimmer	Metallic
Titchener et al., 2015, United Kingdom [36]	Prospective	73(86)	60.3 ± 11.25	59/14	33.0 ± 11.25	ToeFit-Plus	Metallic
Tunstall et al., 2017, United Kingdom [37]	Prospective	30(33)	58.6 ± 8.0	21/9	16.9 ± 4.25	Roto-Glide	Metallic
Wassink et al., 2016, Netherlands/Spain [38]	Prospective	53(58)	58 ± 11.75	NR	24.0 ± NR	Metis (Integra)	Metallic
Wetke et al., 2012, Denmark [39]	Prospective	12(12)	56 ± 3.5	3/9	37.2 ± 18.61	Roto-Glide	Metallic

NR = not reported

### Risk of bias assessment

**Fig. 2:**
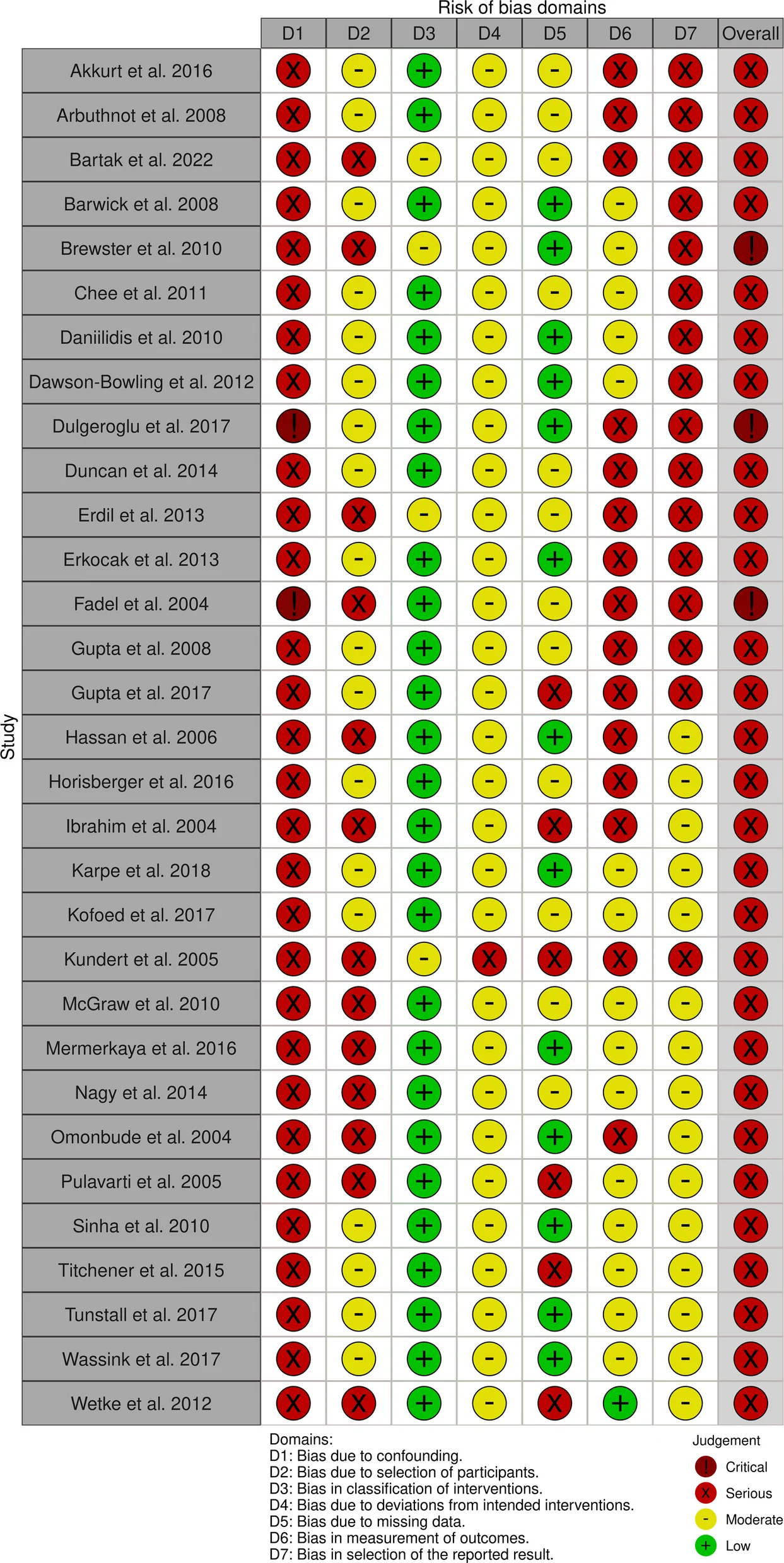
ROBINS-I evaluation

**Fig. 3:**
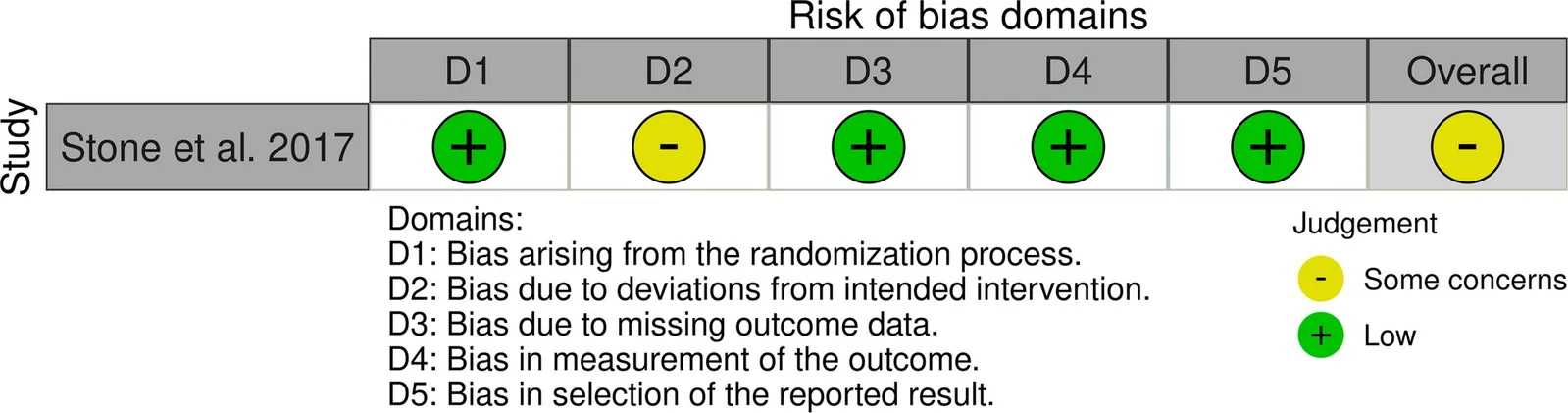
RoB 2 evaluation

Risk-of-bias assessment showed that most included non-randomised studies were at overall serious or critical risk of bias, driven mainly by participant selection, lack of comparators, and inconsistent outcome measurement and reporting. Bias due to confounding was frequently serious or critical, reflecting the predominance of single-arm retrospective and prospective cohort designs (Fig. 2). The single randomised study was assessed separately using RoB 2 (Fig. 3). Overall, the evidence base was judged to be methodologically limited, and these findings were considered when interpreting the pooled estimates.

### Overview of quantitative synthesis

Patient-reported outcome measures (PROMs) were synthesised separately by outcome domain to avoid pooling non-equivalent measures. The primary pooled outcomes were AOFAS-HMI, VAS, and ROM, representing function, pain, and motion, respectively. For each study, a single postoperative timepoint was selected according to the prespecified rule, using the timepoint closest to 24 months where explicitly reported, or studies with mean follow-up between 12 and 36 months where discrete postoperative timepoints were not stated. Sensitivity analyses were then undertaken to assess the robustness of the pooled estimates to broader inclusion assumptions, including studies with longer follow-up. A summary of the primary pooled PROM meta-analyses is presented in Table 2.

**Table 2:** Pooled PROM results

Outcome	Studies (k)	Pooled Estimate	95% CI	*p*-value	Q	I^2^	τ^2^	95% Prediction Interval
AOFAS-HMI	12	42.76	38.44 to 47.09	< 0.001	147.52	91.0%	41.68	27.91 to 57.61
VAS	7	5.24	3.82 to 6.67	< 0.001	327.66	98.3%	2.29	1.28 to 9.21
ROM	13	27.02	17.19 to 36.86	< 0.001	1398.20	99.0%	261.50	− 9.56 to 63.61

### Functional outcomes: AOFAS-HMI

**Fig. 4:**
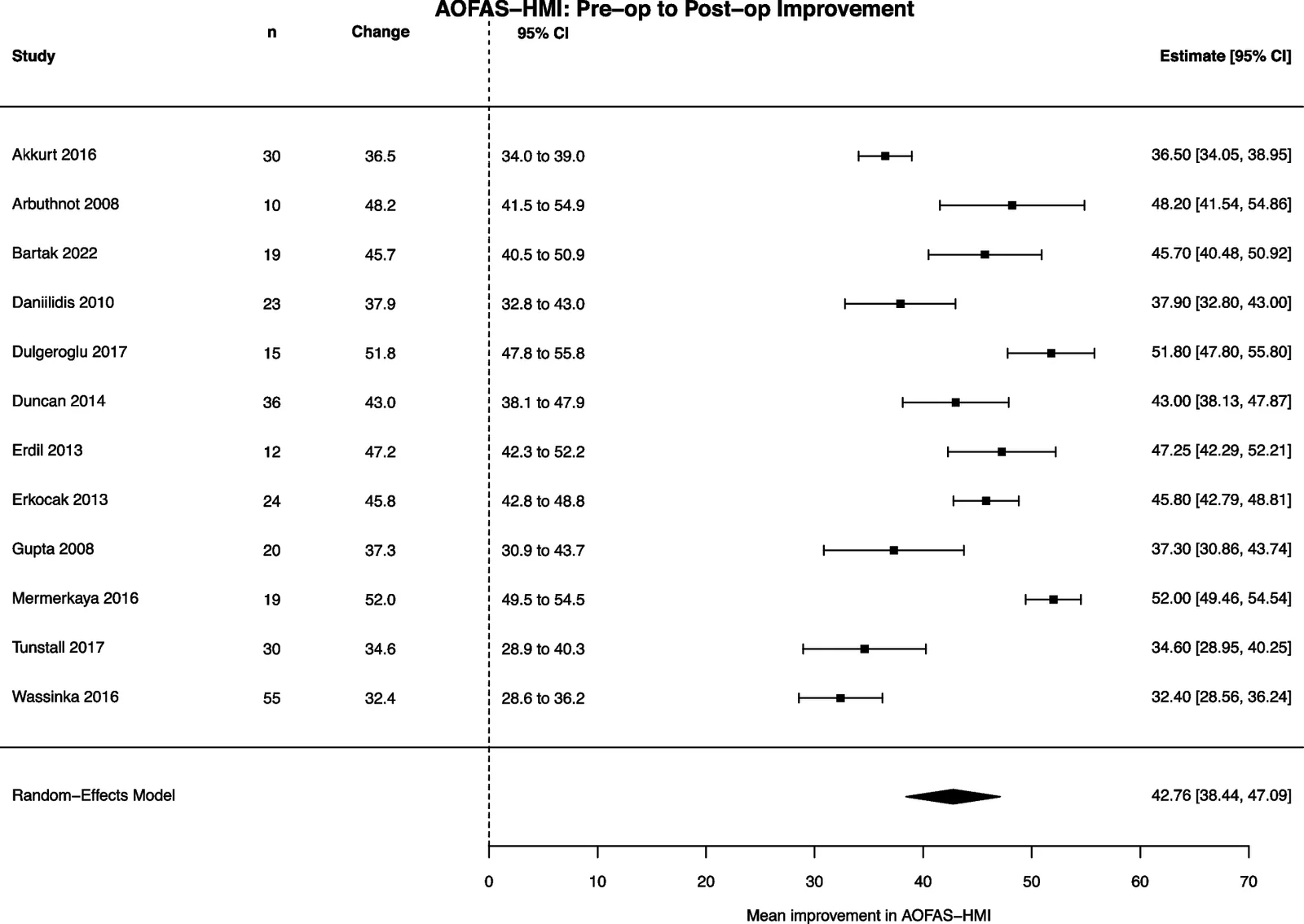
Forest plot of AOFAS-HMI

Twelve studies were included in the primary AOFAS-HMI meta-analysis. In a random-effects model assuming a pre-post correlation of 0.5, total first MTP arthroplasty was associated with a pooled improvement of 42.76 points (95% CI 38.44–47.09, *p* < 0.001; Fig. 4).

Between-study heterogeneity was high (Q = 147.52, *p* < 0.001; I^2^ = 91.0%, τ^2^ = 41.68). However, the 95% prediction interval remained entirely positive (27.91 to 57.61; Table 2), indicating that functional improvement would still be expected across a range of comparable clinical settings despite substantial between-study variability.

The result was stable across assumed pre-post correlations (*r* = 0.3, 0.5, and 0.7), with negligible change in the magnitude of effect, supporting the robustness of the finding to this assumption.

Leave-one-out analyses showed that no individual study materially altered the overall result, with all leave-one-out pooled estimates remaining positive and of similar magnitude. Diagnostic analyses identified a small number of studies contributing more strongly to heterogeneity than others, but no study exerted disproportionate influence sufficient to change the overall interpretation.

Sensitivity analyses yielded similar findings. Inclusion of broader-follow-up and less comparable cohorts reduced the pooled estimate modestly to approximately 39.9 to 40.7 points, but the direction and clinical magnitude of effect were unchanged. These models were associated with increased heterogeneity, reflecting reduced comparability of included studies, but did not alter the overall conclusion.

Overall, AOFAS-HMI findings indicate a large and clinically meaningful postoperative improvement following arthroplasty, with robustness across modelling assumptions and sensitivity analyses despite substantial between-study heterogeneity.

### Pain outcomes: VAS

**Fig. 5:**
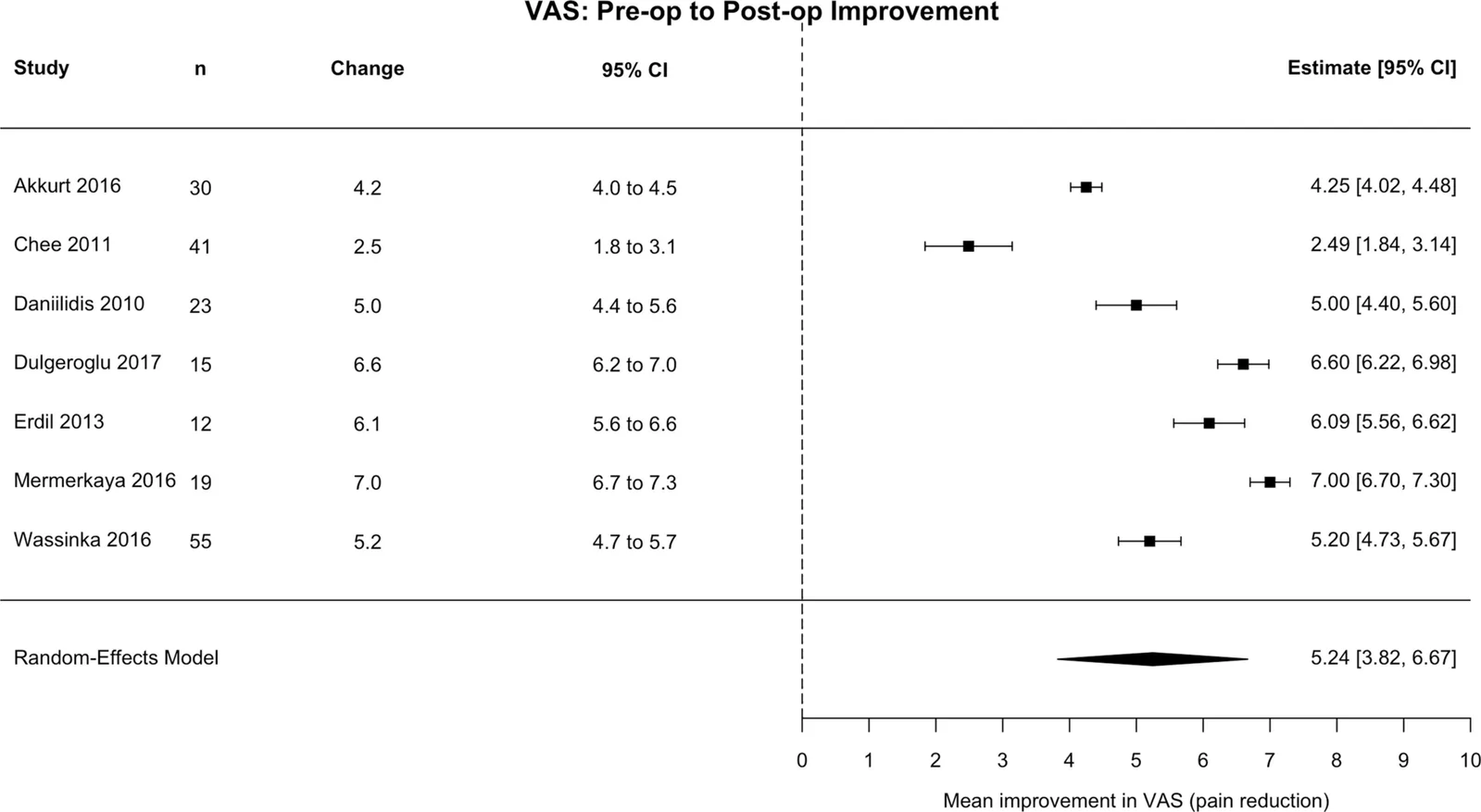
Forest plot of VAS score

Seven studies were included in the primary VAS meta-analysis (Table 2). In a random-effects model assuming a pre-post correlation of 0.5, total first MTP arthroplasty was associated with a pooled reduction in VAS pain score of 5.24 points (95% CI 3.82–6.67, *p* < 0.0001; Fig. 5).

Between-study heterogeneity was very high (Q = 327.66, *p* < 0.001; I^2^ = 98.3%, τ^2^ = 2.29). Despite this, the 95% prediction interval remained entirely positive (1.28 to 9.21; Table 2), indicating that pain reduction would still be expected across a range of comparable clinical settings despite substantial between-study variability.

The result was stable across assumed pre-post correlations (*r* = 0.3, 0.5, and 0.7), with negligible change in the magnitude of effect, supporting the robustness of the finding to this assumption.

Leave-one-out analyses showed that omission of any individual study did not materially alter the overall result, with all leave-one-out pooled estimates remaining positive and of similar magnitude. Diagnostic analyses identified one study as contributing more strongly to heterogeneity than the others, but the overall interpretation of substantial postoperative pain reduction was unchanged.

Sensitivity analyses yielded similar findings. Inclusion of broader-follow-up and less comparable cohorts had minimal effect on the pooled estimate, which remained approximately 5.1 to 5.2 points, while the direction and clinical magnitude of effect were unchanged. These analyses therefore supported the robustness of the primary pain outcome despite persistent heterogeneity.

Overall, VAS findings indicate a large and clinically meaningful postoperative reduction in pain following arthroplasty, with strong robustness across modelling assumptions and sensitivity analyses despite substantial between-study heterogeneity.

### Motion outcomes: ROM

**Fig. 6:**
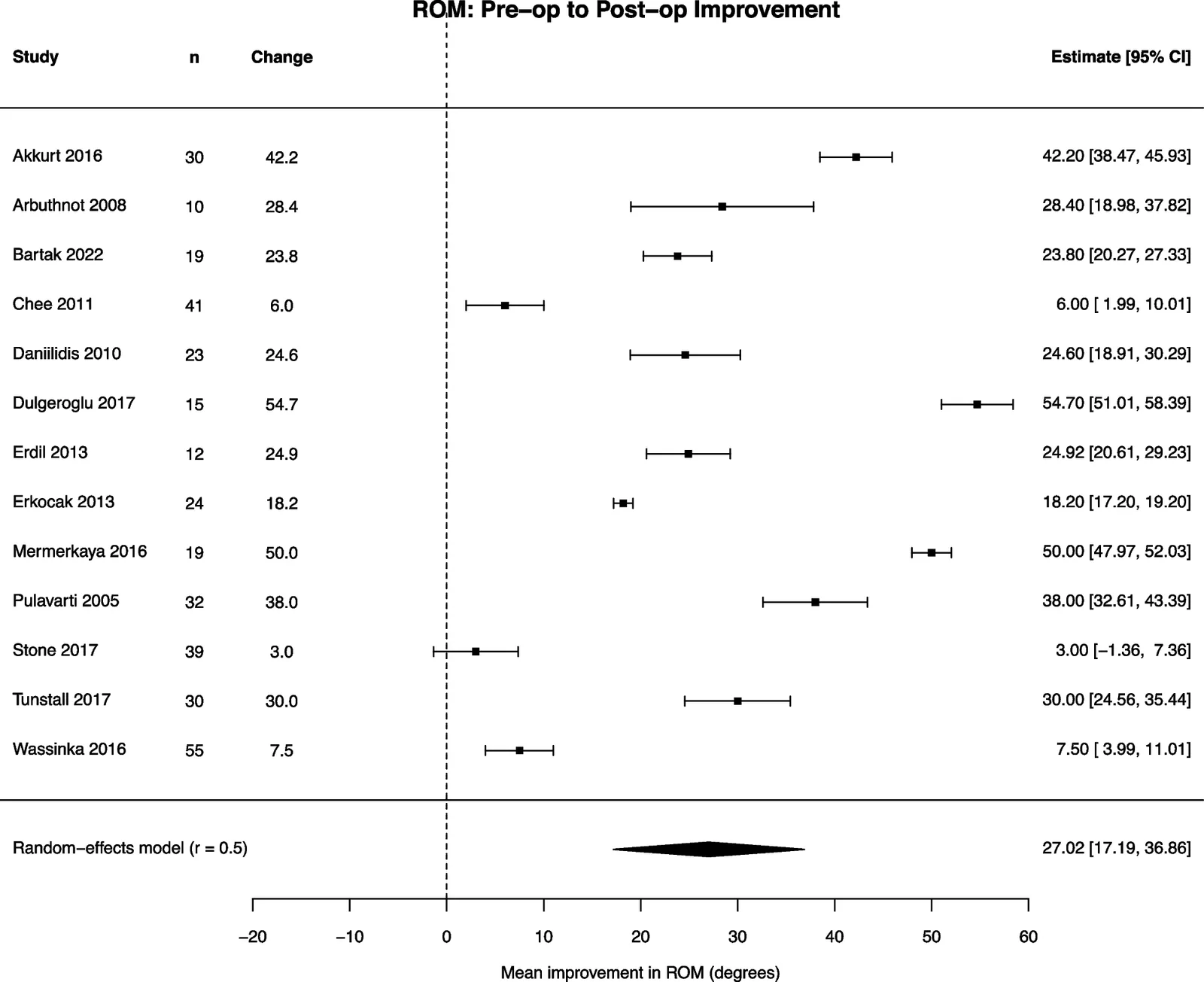
Forest plot of ROM

Thirteen studies were included in the primary ROM meta-analysis (Table 2). In a random-effects model assuming a pre-post correlation of 0.5, total first MTP arthroplasty was associated with a pooled improvement in ROM of 27.02 degrees (95% CI 17.19–36.86, *p* < 0.001; Fig. 6).

Between-study heterogeneity was extreme (Q = 1398.20, *p* < 0.001; I^2^ = 99.0%, τ^2^ = 261.50). Unlike the AOFAS-HMI and VAS analyses, the 95% prediction interval crossed zero (− 9.56 to 63.61; Table 2), indicating that although the average pooled effect was positive, the magnitude of ROM improvement was highly inconsistent across studies and may be minimal in some comparable clinical settings.

The pooled estimate remained stable across assumed pre-post correlations (*r* = 0.3, 0.5, and 0.7), with little change in the magnitude of effect, suggesting that the main limitation of this synthesis was not the correlation assumption but the severity of between-study heterogeneity.

Leave-one-out analyses showed that omission of any individual study did not reverse the overall finding of postoperative ROM improvement, with all leave-one-out pooled estimates remaining positive and of similar magnitude. Diagnostic analyses identified several studies contributing more strongly to heterogeneity than others, but no individual study materially altered the overall interpretation.

Sensitivity analyses yielded similar findings. Inclusion of an additional less comparable cohort reduced the pooled estimate modestly to 24.47 degrees (95% CI 13.90–35.05) and increased heterogeneity (I^2^ = 99.2%, τ^2^ = 331.25; Table 3). The prediction interval remained wide and crossed zero. Thus, although the average direction of effect remained positive, ROM outcomes were substantially less consistent and less generalisable than the corresponding pain and functional score findings.

**Table 3:** Summary of sensitivity analyses

Model	Studies (k)	Pooled Estimate	95% CI	*p*-value	Q	I^2^	τ^2^	95% Prediction Interval
AOFAS-HMI	12	42.76	38.44 to 47.09	< 0.001	147.52	91.0%	41.68	27.91 to 57.61
AOFAS + McGraw	13	40.71	34.78 to 46.64	< 0.001	243.39	95.5%	90.25	19.18 to 62.24
AOFAS + McGraw + Horisberger	14	39.88	34.13 to 45.63	< 0.001	261.06	95.4%	92.79	18.29 to 61.47
VAS main	7	5.24	3.82 to 6.67	< 0.001	327.66	98.3%	2.29	1.28 to 9.21
VAS + Wetke	8	5.17	3.96 to 6.39	< 0.001	329.05	97.8%	2.04	1.58 to 8.76
VAS + Horisberger	8	5.18	3.98 to 6.38	< 0.001	331.41	97.8%	2.00	1.63 to 8.73
VAS + Wetke + Horisberger	9	5.12	4.07 to 6.18	< 0.001	332.70	97.3%	1.81	1.85 to 8.40
ROM main	13	27.02	17.19 to 36.86	< 0.001	1398.20	99.0%	261.50	− 9.56 to 63.61
ROM + Horisberger	14	24.47	13.90 to 35.05	< 0.001	1555.92	99.2%	331.25	− 16.24 to 65.19

Overall, ROM findings suggest a positive average postoperative gain in motion following arthroplasty, but with markedly greater between-study variability and uncertainty than observed for AOFAS-HMI or VAS.

### Comparative interpretation across PROM domains

Taken together, the primary meta-analyses showed that total first MTP arthroplasty was associated with substantial postoperative improvement across pain, function, and motion outcomes. AOFAS-HMI and VAS both demonstrated large, pooled improvements with wholly positive prediction intervals, supporting greater confidence that functional improvement and pain reduction would be expected across comparable clinical settings (Table 2).

In contrast, ROM also improved on average, but showed markedly greater heterogeneity and a prediction interval crossing zero (Table 2). This indicates that although postoperative motion gain was positive overall, the magnitude of benefit was much less consistent and less generalisable than the improvements in pain and function.

Accordingly, the contribution of the quantitative synthesis was not to confirm postoperative improvement, but to demonstrate that pain relief and functional improvement are more consistently supported by the available evidence than restoration of motion.

### Sensitivity analyses

Across all three outcome domains, the direction of effect remained unchanged, indicating that the primary findings were robust to broader inclusion assumptions. However, the impact of these additional studies differed by outcome.

For AOFAS-HMI, inclusion of broader-follow-up and less comparable cohorts reduced the pooled estimate from 42.76 points in the main model to 40.71 points with the inclusion of McGraw et al. and to 39.88 points with the additional inclusion of Horisberger et al. In all models, the pooled estimate remained large and clearly positive. However, heterogeneity increased substantially, with I^2^ rising from 91.0% in the primary analysis to approximately 95.4%–95.5% in the broader models and τ^2^ more than doubling. The prediction interval remained wholly positive in all AOFAS-HMI sensitivity models.

For VAS, the pooled estimate was highly stable. The addition of the broader-follow-up Wetke cohort, the Horisberger cohort, or both changed the pooled estimate only minimally, from 5.24 points in the main model to values between 5.12 and 5.18 points. Confidence intervals remained clearly positive throughout. Although heterogeneity remained very high in all models, τ^2^ fell slightly with broader inclusion, and the prediction interval remained entirely positive. These findings indicate that the pain outcome was particularly robust to the sensitivity analyses undertaken.

For ROM, inclusion of Horisberger et al. reduced the pooled estimate from 27.02 to 24.47 degrees. The confidence interval remained positive, but heterogeneity increased further (I^2^ 99.2%, τ^2^ 331.25), and the prediction interval continued to cross zero. Thus, while the direction of effect remained positive, ROM remained the least stable and least generalisable of the three pooled outcome domains.

Taken together, these sensitivity analyses support the use of the primary models as the main quantitative results. Broader inclusion of less comparable studies did not reverse any of the pooled findings, but in some instances, particularly for AOFAS-HMI and ROM, it increased heterogeneity and reduced comparability.

### Secondary descriptive outcomes: revision, survivorship, and complications

Revision, survivorship, and complication outcomes were extracted to contextualise the PROM findings, but were not pooled quantitatively because reporting definitions, denominators, and follow-up intervals were heterogeneous. Accordingly, the figures presented below represent crude observational frequencies calculated from the total number of reported events and feet. They are not annualised incidence rates, Kaplan–Meier survivorship estimates, or formal meta-analytic pooled proportions. A summary of these secondary outcomes is presented in Table 4.

**Table 4:** Secondary crude descriptive revision, survivorship, and complication outcomes across variably followed cohorts

Variable	Events/reported feet, crude frequency
Revision events/reported feet	101/1008 (10.0%)
Loosening events/reported feet	84/973 (8.63%)
Infection events/reported feet	19/973 (1.95%)
Implant failure events/reported feet	55/936 (5.71%)
Persistent pain events/reported feet	62/860 (7.21%)
Non-revision reoperations/reported feet	25/759 (3.29%)
Radiographic failures/reported feet	130/894 (14.5%)

Across the extracted survivorship dataset, 32 study entries contributed revision and survival information, representing 1,008 operated feet. A total of 101 revisions were reported, corresponding to a crude revision proportion of 10.0% across all reported feet. This crude proportion should be interpreted cautiously, as revision definitions and follow-up duration varied substantially between studies. Kaplan–Meier survivorship data were reported inconsistently and at non-uniform time points, precluding meaningful pooled synthesis within this PROMs-focused review.

Complication reporting was similarly variable. Descriptively, across studies reporting each complication category, the extracted dataset recorded 84 loosening events across 973 reported feet, 19 infections across 973 feet, 55 implant failures across 936 feet, 62 cases of persistent pain across 860 feet, 25 non-revision reoperations across 759 feet, and 130 radiographic failures across 894 feet (Table 4). These values are presented as descriptive totals only and should not be interpreted as pooled complication rates, given the inconsistency in event definitions, reporting thresholds, and denominators.

Overall, the secondary outcome data suggest that postoperative PROM improvement should be interpreted alongside heterogeneous reporting of revision and complication outcomes. While these findings provide important clinical context, the available survivorship and safety data were less standardised and less directly comparable than the primary PROM outcomes.

## Discussion

### Overall interpretation

This systematic review and meta-analysis found that total first metatarsophalangeal (MTP) joint arthroplasty was associated with substantial postoperative improvement in pain and function at medium-term follow-up, whereas improvement in range of motion (ROM) was less consistent.

In pooled within-group analyses, total first MTP arthroplasty was associated with a mean improvement of 42.8 points in AOFAS-HMI (95% CI 38.4–47.1) and a 5.2-point reduction in VAS pain (95% CI 3.8–6.7), both large and clinically meaningful effects, whereas the pooled ROM gain of 27.0 degrees (95% CI 17.2–36.9) showed much greater study variability.

The principal contribution of this synthesis is therefore not simply to confirm that patients improve after arthroplasty, but to demonstrate that the evidence is stronger and more consistent for symptom relief and functional improvement than for reliable restoration of motion.

These findings should be interpreted within the context of the wider comparative literature. Two meta-analyses comparing arthroplasty with arthrodesis have suggested that overall clinical outcomes are often similar; pain relief tends to favour arthrodesis in comparative meta-analyses [4, 8]. This signal persisted at longer-term follow-up, with Stone et al. reporting less pain, greater satisfaction, and fewer revisions after arthrodesis at 15 years [35]. Accordingly, the present review supports total first MTP arthroplasty as a valid motion-preserving option in selected patients, but it does not challenge the position of arthrodesis as the most predictable benchmark against which replacement procedures continue to be judged.

A further important interpretive point is that arthroplasty cannot be understood as a single, uniform intervention. The literature included in this review spans multiple implant families, generations, materials, and fixation strategies, and these differences are highly likely to influence outcome. The pooled estimates are therefore clinically meaningful, but they should be understood as mixed-technology averages rather than the performance of any one implant platform. Although PROMs were generally favourable, these findings were offset by significant revision and radiographic failure proportions, and by inconsistency in restoration of postoperative ROM.

### Functional outcomes and pain relief

The strongest findings in the present review were seen for AOFAS-HMI and VAS. Arthroplasty was associated with a large, pooled improvement in function and a substantial reduction in pain, and these findings remained directionally stable in sensitivity analyses. Clinically, this is important because pain relief is the primary indication for surgery in many patients with advanced hallux rigidus.

The included cohort literature is broadly consistent with this result. Daniilidis et al. reported improvement in AOFAS from 44.6 to 82.5 and VAS from 7.0 to 2.0 after ToeFit-Plus total replacement at 18 months [15]. Dulgeroglu and Metineren reported AOFAS improvement from 26.9 to 78.7 and VAS improvement from 8.3 to 1.7 after total arthroplasty [17]. Erkocak et al. similarly found mean AOFAS improvement from 42.7 to 88.5 and pain improvement from 7.4 to 1.9 in a prospective ToeFit-Plus series [6]. Wassink et al. reported significant improvement in AOFAS-HMI and pain at 24 months with a three-component press-fit prosthesis [38]. Arbuthnot et al. and Mermerkaya and Adli also described clear postoperative functional improvement in ceramic total cohorts [10, 30].

However, these within-group improvements should not be conflated with proof of superiority over fusion. The present review provides strong evidence for the former, but not for the latter. The best comparative evidence continues to suggest that arthroplasty may improve symptoms substantially, yet arthrodesis remains more reliable for pain relief [4, 8, 35]. The distinction between “improved from baseline” and “superior to arthrodesis” is therefore crucial when interpreting the arthroplasty literature. Total first MTP arthroplasty should therefore be discussed as a procedure that can achieve meaningful symptomatic improvement, rather than one that has clearly surpassed arthrodesis as the standard treatment for advanced disease.

### Range of motion

ROM was the least consistent outcome domain in this review. Although the pooled estimate suggested a positive average postoperative gain of 27.02°, between-study heterogeneity was extreme, and the prediction interval crossed zero, indicating that clinically meaningful motion gain may not be reproducible across all comparable settings.

The included studies illustrate this variability: Daniilidis et al. reported improvement in total ROM from 28.1° to 52.7° [15], Erkocak et al. from 25.9° to 53.8° [6], Akkurt et al. from 17° to 59° [9], and Bartak et al. from approximately 14–15° to 32° at long-term follow-up [11], while Pulavarti’s Bio-Action cohort also demonstrated improved dorsiflexion and total arc [33]. By contrast, Arbuthnot et al. found that ROM declined over time after initially favourable early results, reaching 33.3° at 24 months [10], Horisberger et al. reported intraoperative improvement followed by reduction to 29.0° at final follow-up despite retained pain relief in surviving implants [24], Chee et al. showed only a small, non-significant increase from 28° to 34° [14], and Dawson-Bowling et al. found that more than half of unrevised joints had less than 36° of motion, with only one joint exceeding 60° [16]. Wassink et al. also reported improved dorsiflexion, but this was accompanied by reoperations and radiographic abnormalities in a subset of cases [38].

Taken together, these findings suggest that arthroplasty may preserve or restore a clinically useful residual arc of motion in many patients, but does not reliably restore physiologic movement, and the magnitude and durability of ROM gain appear to depend on implant design, pre-existing stiffness, surgical technique, and rehabilitation. As preservation of motion remains the principal theoretical advantage of arthroplasty over arthrodesis, this distinction is important in practice: retained first-MTP mobility may be valuable for footwear choice, kneeling, squatting, or perceived gait naturalness, but it should be framed in preoperative counselling as a potential, rather than guaranteed, benefit. These functional effects may not be fully captured by conventional PROMs or static ROM measurements, and objective gait assessment may therefore provide additional insight into the functional impact of hallux rigidus and its treatment [40].

### Complications, loosening and revision

The findings relating to complications and revision help explain much of the heterogeneity observed in the pooled analyses. Comparative meta-analyses have not consistently demonstrated a clear difference in revision or complication rates between arthroplasty and arthrodesis. However, the implant-specific literature included in this review shows that durability varies markedly between prosthesis families, indicating that pooled arthroplasty estimates may obscure clinically important differences. Recognised complications of first MTP arthroplasty across the included studies included radiolucency, loosening, implant failure, persistent pain, infection, subsidence, malalignment, and radiographic failure. Across the 32 included studies, radiographic failure was documented in 14.5% of evaluable implants, the overall crude revision proportion was 10.0% of reported feet, loosening occurred in 8.6%, infection in 2.0%, and persistent pain in 7.2%, although these figures should be interpreted cautiously because definitions, denominators, and follow-up durations varied substantially. Furthermore, variation in follow-up duration may have led to underestimation of late implant complications, as shorter-term cohorts may not capture progressive radiolucency, aseptic loosening, implant wear, osteolysis, or subsequent revision that becomes evident only with longer surveillance.

ToeFit-Plus illustrates this pattern clearly. Early and medium-term studies by Daniilidis, Duncan, and Erkocak reported encouraging short-term gains in pain and function, but longer-term follow-up was much less reassuring [6, 15, 18]. Gupta and Masud reported a 21% failure or revision rate at a mean follow-up of 134 months and concluded that the implant should not be recommended, while Titchener et al. reported a 24% revision rate at a mean of 33 months and discontinued use of the implant [22, 36]. Bartak et al. similarly found high long-term revision rates and concluded that their unit no longer used the system because of its failure profile [11].

The ceramic MOJE literature showed a comparable trajectory: early small studies reported favourable short-term results, but later studies documented worsening durability, with Dawson-Bowling et al. reporting radiographic loosening in over half of cases and a reoperation rate of 26%, McGraw et al. finding widespread loosening and subsidence at a mean of 44 months despite acceptable mid-term clinical scores [16, 29], and Nagy et al. reporting poor clinical and radiological outcomes with a 16% revision rate and implant survival falling to 68% at 9 years [31].

In contrast, not all implant systems showed poor durability: Kofoed et al. reported more favourable long-term results with Rotoglide, including no aseptic loosening and 91.5% survival at 15 years [27], while Karpe et al. described encouraging early to mid-term outcomes with the same implant [26].

Biomechanical factors may also influence implant durability. First-ray instability and hindfoot valgus can alter loading across the first MTP joint and should therefore be considered during preoperative assessment and patient selection. Although direct evidence linking these factors to prosthetic loosening or failure remains limited, uncorrected malalignment may plausibly contribute to adverse mechanical loading of the implant [41].

Taken together, these findings indicate that there is no single revision profile for first-MTP arthroplasty; rather, durability is highly implant-specific and appears to decline in some systems with longer follow-up. This has clear implications for practice, as the complication discussion should address not only whether the primary procedure improves symptoms, but also the consequences of failure. Failed arthroplasty may leave bone loss, subsidence, malalignment, or soft tissue compromise, making salvage more complex than primary fusion, and this is particularly relevant when counselling younger or more active patients who may face a greater cumulative lifetime risk of revision.

### Limitations and future directions

This review has several important limitations, most of which reflect the quality and heterogeneity of the available evidence base. The included studies were predominantly Level III-IV evidence, consisting mainly of retrospective case series and single-arm prospective cohorts, with only one RCT. As a result, causal inference is limited, and the findings are more vulnerable to selection bias and confounding. ROBINS-I assessment identified serious or critical risk of bias across much of the included literature, driven largely by single-centre, single-surgeon designs, variable patient selection, and inconsistent outcome reporting. These pooled improvements should therefore be interpreted cautiously, as uncontrolled observational studies may overestimate treatment effects through selection bias, regression to the mean, and incomplete outcome reporting. Consequently, the pooled estimates should not be used to support routine arthroplasty or to recommend a specific implant system. Rather, they provide low-certainty evidence that carefully selected patients may experience postoperative improvement. Surgical decision-making should therefore remain individualised, with arthrodesis retained as a more predictable benchmark treatment, with arthroplasty considered only after discussion surrounding implant durability uncertainty, revision risk, and likelihood of achieving meaningful preservation of motion.

A further methodological limitation concerns potential within-study non-independence arising from bilateral procedures. The quantitative synthesis used the outcome-specific sample available at the selected postoperative timepoint, which was often smaller than the original cohort because of loss to follow-up and incomplete outcome reporting. In addition, several studies did not report how many bilateral cases contributed to individual outcomes. A sensitivity analysis excluding bilateral procedures was therefore not feasible due to inconsistent reporting.

Heterogeneity was also substantial with respect to implant type, implant generation, follow-up interval, and reporting method, making pooled estimates clinically informative but methodologically blunt. However, these potential sources of heterogeneity could not be examined reliably through subgroup analysis or meta-regression. Ceramic implants were represented in only 2 of 12 AOFAS-HMI studies, 2 of 7 VAS studies, and 3 of 13 ROM studies. Consequently, stratified pooled estimates would have been imprecise, underpowered, and potentially confounded. The pooled estimates should be interpreted as mixed-technology averages rather than as expected outcomes for any specific implant material or prosthesis design.

Definitions of survivorship and failure varied considerably, with some studies reporting implant retention, others any reoperation, and others only conversion to fusion. Selection effects were especially important, as the more favourable arthroplasty cohorts often used relatively strict indications. For example, Karpe et al. limited arthroplasty to patients with advanced disease, good bone stock, and acceptable alignment, excluding inflammatory arthritis, significant hallux valgus, neurovascular compromise, and poor local tissue conditions [26]; Daniilidis et al. excluded severe deformity, previous forefoot surgery, and diabetes [15]; and Wassink et al. excluded marked valgus, instability, avascular necrosis, and advanced vascular disease [38]. These criteria indicate that the present findings apply most directly to carefully selected arthroplasty candidates rather than to all patients with advanced hallux rigidus.

Taken together, the available evidence suggests that total first MTP arthroplasty is most appropriately considered in older or lower-demand patients with end-stage hallux rigidus who prioritise preservation of joint motion for footwear choice or lower-impact activities requiring MTP dorsiflexion. The ideal candidate should have adequate bone stock, satisfactory vascularity and soft-tissue condition, stable first-ray mechanics, acceptable forefoot and hindfoot alignment, and minimal fixed deformity. By contrast, arthrodesis is likely to remain the more predictable option for younger or high-demand patients, those requiring durable stability for high-impact activity or occupational footwear, and patients with substantial deformity, instability, neuropathy, severe osteoporosis or other factors likely to compromise implant fixation and survivorship.

Finally, longer-term evidence remains sparse for several implant families, particularly newer designs, and this is important because a number of systems showed favourable early outcomes but worsening survivorship with extended follow-up. Interpretation of total first MTP arthroplasty efficacy must therefore consider not only the magnitude of early postoperative improvement, but also the time over which benefit is assessed.

Future research should prioritise implant-specific prospective comparative studies, standardised definitions of survivorship and failure, and longer follow-up for contemporary implant designs.

## Conclusion

Total first metatarsophalangeal (MTP) joint arthroplasty is associated with substantial improvement in pain and function, but current evidence does not support uniformly reliable implant performance. Revision and radiographic failure rates remain appreciable, while postoperative ROM, although improved on average, is inconsistent and does not reliably restore physiological movement. These findings suggest that, despite favourable PROMs in some cohorts, contemporary total first MTP arthroplasty remains limited by durability concerns and non-physiological motion restoration. Arthrodesis therefore remains the benchmark against which total joint replacement should be judged. Further development of implant design, high-quality prospective studies with standardised outcome reporting, and long-term follow-up are needed to define the role of contemporary total first MTP arthroplasty more clearly.

## Supplementary Information

Supplementary Material 1.

Supplementary Material 2.

## Data Availability

Data supporting the findings of this study are available from the corresponding author upon reasonable request.
