# Supplementary material for: Outcomes after total first metatarsophalangeal joint arthroplasty for hallux rigidus: a systematic review and meta-analysis
Source: Arthroplasty. 2026 Oct 2;8:73. doi: 10.1186/s42836-026-00433-3 (PMC13632088; doi:10.1186/s42836-026-00433-3)
Supplement: Supplementary file 1 [file 42836_2026_433_MOESM1_ESM.pdf]

Citation	Outcome Measure	Timepoint	Pre-Op Mean	Pre-Op SD	Post-Op Mean	Post-Op SD	Sample Size for PROM
Akkurt et al., 2016	ROM (degrees)	Final followup	17.2	6.8	59.4	12	30
Akkurt et al., 2016	AOFAS-HMI	Final followup	41.5	6.86	78	6.86	30
Akkurt et al., 2016	VAS	Final followup	7.25	0.74	3	0.49	30
Arbuthnot et al., 2008	ROM (degrees)	24mo	4.9	5.52	33.3	17.19	10
Arbuthnot et al., 2008	AOFAS-HMI	24mo	36	10.79	84.2	10.69	10
Bartak et al., 2022	ROM (degrees)	24mo	13.6	5.4	37.4	9	19
Bartak et al., 2022 (TA)	AOFAS-HMI	24mo	38.2	12.4	83.9	10.6	19
Chee et al. 2011	ROM (degrees)	Final followup	28	14	34	12	41
Chee et al. 2011	VAS	mean 3+/-2 years	5.15	1.85	2.66	2.33	41
Daniilidis et al. 2010	AOFAS-HMI	18 months	44.6	7.2	82.5	14.4	23
Daniilidis et al. 2010	VAS	18 months	7	0.8	2	1.7	23
Daniilidis et al. 2010	ROM (degrees)	18 months	28.1	4.9	52.7	15.7	23
Dulgeroglu et al. 2017	AOFAS-HMI	Final followup	26.9	2.3	78.7	8.8	15
Dulgeroglu et al. 2017	VAS	Final followup	8.3	0.8	1.7	0.7	15
Dulgeroglu et al. 2017	ROM (degrees)	Final followup	22.3	7.7	77	6.8	15
Duncan et al. 2014	AOFAS-HMI	2 years	39	6.1	82	17	36
Erdil et al. 2013 (TA)	AOFAS-HMI	Final followup	45.42	9.69	92.67	7.39	12
Erdil et al. 2013	VAS	Final followup	7.67	1.07	1.58	0.67	12
Erdil et al. 2013	ROM (degrees)	Final followup	15.08	4.03	40	8.79	12
Erkocak et al. 2013	AOFAS-HMI	24 mo	42.7	5.75	88.5	8.5	24
Erkocak et al. 2013	ROM (degrees)	24 mo	10.4	2.5	28.6	2.5	24
Gupta et al. 2008	AOFAS-HMI	Final followup	39.4	12	76.7	16.4	20
Mermerkaya et al. 2016	ROM (degrees)	27.1 months	25	5	75	3.75	19
Mermerkaya et al. 2016 (Group II -	AOFAS-HMI	27.1 months	31	6.25	83	4.75	19
Mermerkaya et al. 2016	VAS	27.1 months	9	0.75	2	0.5	19
Pulavarti et al. 2005	ROM (degrees)	36 months	6	5.25	44	17.5	32
Stone et al. 2017	ROM (degrees)	24 months	11	7	14	16	39
Tunstall et al. 2017	AOFAS-HMI	Final followup	41.4	12.5	76	17.75	30
Tunstall et al. 2017	ROM (degrees)	Final followup	31	10	61	17.5	30
Wassinka et al., 2016	AOFAS-HMI	24 mo	49.6	15.1	82	13.9	55
Wassinka et al., 2016	VAS	24 mo	6.8	1.6	1.6	1.9	55
Wassinka et al., 2016	ROM (degrees)	24 mo	26.9	12.8	34.4	13.7	55
