# Supplementary material for: Outcomes after total first metatarsophalangeal joint arthroplasty for hallux rigidus: a systematic review and meta-analysis
Source: Arthroplasty. 2026 Oct 2;8:73. doi: 10.1186/s42836-026-00433-3 (PMC13632088; doi:10.1186/s42836-026-00433-3)
Supplement: Supplementary file 2 [file 42836_2026_433_MOESM2_ESM.pdf]

Supplementary Material

1.0 DATABASE SEARCH

The final database search was conducted on 4 May 2025 and covered publications from January 1990 to the date of search.

MEDLINE & EMBASE (OVID)

Line	Search string
1	"first metatarsophalangeal joint".mp.
2	"first MTPJ".mp.
3	hallux.mp.
4	"big toe".mp.
5	"first toe".mp.
6	1 or 2 or 3 or 4 or 5
7	"hallux rigidus".mp.
8	osteoarthritis.mp.
9	7 or 8
10	arthroplasty.mp.
11	"total joint replacement".mp.
12	prosthesis.mp.
13	implant.mp.
14	10 or 11 or 12 or 13
15	6 and 9 and 14

COCHRANE LIBRARY

Line	Search string
1	"first metatarsophalangeal joint" OR "first MTPJ" OR hallux OR "big toe" OR "first toe"
2	"hallux rigidus" OR osteoarthritis
3	arthroplasty OR "total joint replacement" OR prosthesis OR implant
4	#1 AND #2 AND #3

2.0 CHANGE-SCORE STANDARD DEVIATIONS AND CORRELATION ASSUMPTIONS

For all study-outcome estimates included in the quantitative syntheses, the standard deviation of the change score was derived from the reported preoperative and postoperative standard deviations using the standard paired-data relationship described in the Cochrane Handbook [1]:

$$SDchange = \sqrt{SD_{pre}^2 + SD_{post}^2 - 2r(SD_{pre})(SD_{post})}$$

where r represents the within-person correlation between preoperative and postoperative measurements. A value of r = 0.5 was applied uniformly across all primary analyses. Sensitivity analyses were repeated using r = 0.3 and r = 0.7. No study-outcome estimate entered the quantitative synthesis using a directly reported SDchange; the derivation approach was therefore identical across all included quantitative estimates.

Sensitivity of pooled estimates to the assumed pre-post correlation

Outcome	r	k	Pooled mean change (95% CI)	I^2 (%)	tau^2	95% prediction interval
AOFAS-HMI	0.3	12	42.79 (38.45 to 47.13)	88.1	40.53	28.12 to 57.46
AOFAS-HMI	0.5	12	42.76 (38.44 to 47.09)	91.0	41.68	27.91 to 57.61
AOFAS-HMI	0.7	12	42.73 (38.42 to 47.05)	94.1	42.91	27.68 to 57.78
VAS	0.3	7	5.25 (3.83 to 6.67)	97.6	2.26	1.31 to 9.19
VAS	0.5	7	5.24 (3.82 to 6.67)	98.3	2.29	1.28 to 9.21
VAS	0.7	7	5.24 (3.82 to 6.66)	98.9	2.33	1.25 to 9.23
ROM	0.3	13	27.03 (17.19 to 36.87)	98.7	260.57	-9.49 to 63.55
ROM	0.5	13	27.02 (17.19 to 36.86)	99.0	261.50	-9.56 to 63.61
ROM	0.7	13	27.02 (17.19 to 36.86)	99.4	262.43	-9.62 to 63.66

REFERENCES

1. Higgins JPT, Li T, Deeks JJ, editors. Chapter 6: Choosing effect measures and computing estimates of effect. In: Higgins JPT, Thomas J, Chandler J, Cumpston M, Li T, Page MJ, Welch VA, editors. *Cochrane handbook for systematic reviews of interventions*. Version 6.5. Cochrane; 2024. Section 6.5.2.8, Imputing standard deviations for changes from baseline.
